# Characterization and Source Identification of Polybrominated Diphenyl Ethers (PBDEs) in Air in Xi’an: Based on a Five-Year Study

**DOI:** 10.3390/ijerph16030520

**Published:** 2019-02-12

**Authors:** Lei Ye, Chengzhong Zhang, Deming Han, Zheng Ji

**Affiliations:** 1 School of Environmental and Municipal Engineering, Xi’an University of Architecture and Technology, Xi’an 710055, Shaanxi, China; yelei@xauat.edu.cn; 2 School of Environmental Science and Engineering, Shanghai Jiao Tong University, Shanghai 200240, China; handeem@sjtu.edu.cn; 3 School of Geography and Tourism, Shaanxi Normal University, Xi’an 710119, Shaanxi, China; 4 International Joint Research Centre of Shaanxi Province for Pollutant Exposure and Eco-Environmental Health, Xi’an 710119, Shaanxi, China

**Keywords:** polybrominated diphenyl ethers, atmosphere, CWT, backward trajectory, Xi’an

## Abstract

In order to assess polybrominated diphenyl ether (PBDE) atmospheric pollution levels in Xi’an, air samples were collected using a large flow air sampler from July 2008 to April 2013. In total, 134 samples were collected and 12 PBDE congeners were detected. Total PBDE concentrations (both gaseous and particulate phase) were 36.38–1054 pg/m^3^, with an average of 253.2 ± 198.4 pg/m^3^. BDE-209 was identified as the main PBDE component, with a corresponding concentration of 0.00–1041 pg/m^3^, accounting for 89.4% of total PBDEs. Principal component analysis results showed that PBDEs in Xi’an’s atmosphere mainly originated from commercial products containing penta-BDE, octa-BDE, and deca-BDE. The relative natural logarithm for partial pressure (RP) of PBDEs (gaseous phase) was calculated using the Clausius–Clapeyron equation. The gas flow trajectories at high, middle, and low RP values were analyzed by applying the backward trajectory model. These data indicated that the difference between trajectory distribution and concentration load on trajectories was huge under different RP values. PBDE concentrations (gaseous phase) weighted trajectory showed that the central and southwestern parts of Henan Province and the northwestern area of Hubei Province exhibited the darkest colors, and the daily average concentration contribution of PBDEs to the receiving point was >9 pg/m^3^, which indicates that these areas might be the main potential source areas of PBDEs in Xi’an’s atmosphere.

## 1. Introduction

Polybrominated diphenyl ethers (PBDEs) are extensively used in the manufacturing process of brominated flame retardants (BFRs), resulting in enhanced flame-retardant properties. Studies have shown that during various manufacturing processes, BFRs are simply physically mixed with other materials with no chemical bonding. This process allows them to easily leach from consumer products into the environment, leading to human exposure and public health threats [1,2].

The first recorded environmental PBDE detection was published in 1979, which identified BDE-209 in soil and sediments near factories producing BFRs in Arkansas, USA [3]. In the following years, several congeners of PBDEs were detected, not only in the environment, but also in organisms such as shuttle fish in the Visken River in Sweden [4]. Since then, research has focused on detecting PBDEs in both abiotic and biotic environmental samples, such as the air, soil, water, sediment, terrestrial and marine organisms, and human samples [5], even in mammalian tissues that have lived in the Baltic sea, North Sea, and the Arctic Ocean [6]. Studies focusing on PBDEs in indoor dust samples indicated extremely heavy pollution and relatively high human exposures, which can cause serious human health burdens [7].

Long-term studies have demonstrated that PBDEs are a very important group of persistent organic pollutants (POPs), which have serious detrimental effects on human health, due to their hydrophobic and lipophilic properties, low vapor pressure, long-term persistence, and high toxicity [8]. There are 10 homologous series, 209 congeners in PBDE homologies, which contain three main types of technical mixtures, specifically “penta-BDE”, “octa-BDE”, and “deca-BDE”. Penta-BDE is liquid and is composed of 36% tetra-BDE (BDE-47, -66), 56% penta-BDE (BDE-99, -100), and 8% hexa-BDE (BDE-153, -154). Octa- and deca-BDE are both solid, the former consists of hepta-BDE (BDE-183), octa-BDE (BDE-197, -203, -196), and bits of hexa-BDEs and nona-BDEs. Meanwhile, deca-BDE is composed of more than 90% BDE-209, with the remaining part mainly consisting of nona-BDEs [9].

In the last thirty years, PBDE-related research has focused on concentration monitoring, source resolution, and gas distribution, etc. These studies show that PBDEs have become ubiquitous in the environment and might be responsible for the synthesis of neurotoxicants, which lead to neurochemical and hormonal deficiencies [10,11,12]. Since 2004, the use of both “penta-BDE” and “octa-BDE” has been banned in the European Union, followed by termination of production by the primary producer in North America [13]. In May 2009, the vast majority of the components comprising these two mixtures, and eight other kinds of POPs, were enlisted during the 4th Conference for the Parties of the Stockholm Convention on POPs held in Geneva [14]. It was declared that a worldwide battle for prohibiting PBDEs has begun. However, “deca-BDEs” are being used worldwide due to their low price and superior performance. Furthermore, their uncertain biological toxicity, especially BDE-209 which accounts for 75% of all BFRs, remains of great concern [15].

Xi’an, the provincial capital of Shaanxi, is one of the most important cities in northwest China. In previously published research, Jiang [16] and Zhou [17] reported concentration distribution characteristics, seasonal variations, and human exposure levels of PBDEs (low bromine) in Xi’an’s atmosphere from the summer of 2008 to the summer of 2009. The data showed that the gas-particle partition coefficient of PBDEs correlated with sub-cooled liquid vapor pressure, and PBDEs pollution levels were most serious in winter compared to any other season. The result of human exposure assessment showed that the inhalation of total BDE-99 by children and adults in Xi’an was below the maximum allowable intake 260 pg/(kg-d) as proposed by De Winter-Sorkina [17,18]. In this context, we aimed to conduct a long-term (5-year) study to monitor PBDE levels in Xi’an’s atmosphere. The main objectives were to obtain the concentration and composition profiles of PBDEs in Xi’an’s atmospheric environment over a long period. Meanwhile, the Hybrid Single-Particle Lagrangian Integrated Trajectory (HYSPLIT) model and a hybrid receptor model (concentration weighted trajectory (CWT)) were applied in order to explore the relationship between the concentration of pollutants and meteorological factors, and to determine the source regions for each pollutant contributing to the corresponding receptor site.

## 2. Materials and Methods

### 2.1. Sample Collection and Preparation

The sampling device was set up on the roof-top of a building at Xi’an University of Architectures and Technology located in the south of Xi’an (34°14′21″ N, 108°57′38″ E), which was above the ground, with no obvious pollution sources and obstacles nearby. The sampling frequency was approximately once per week, commencing the beginning of July 2008 until the end of April 2013, apart from June 2010 to December 2011.

A high-volume air sampler (KB-1000, Kingstar Electronic Technology Co., Ltd., Shenzhen City, GuangDong Province, China) was employed for collecting samples with an average air flowrate of 0.8 m^3^/min for a consecutive 24 h (12:00 a.m. to 12:00 a.m. the next day), which could inhale approximately 1150 m^3^ of air per day. Separate particulate matters and gaseous phase samples were collected using one layer of glass fiber filters (GFF, 20 cm × 25 cm) and two layers of poly urethane foam (PUF) plugs (diameter 9.5 cm, height 5.0 cm). Prior to sampling, GFFs were baked at 450 °C for 7 h and PUF plugs were Soxhlet extracted for 24 h with acetone, and for another 24 h with hexane, to remove any organic contaminant. After sampling, the loaded GFFs were packaged in aluminum foil and stored in a sealed polyethylene bag. The PUFs were kept a in solvent aluminum box twined using polytetrafluoroethylene sintering tape. All samples were transported cold to the Harbin Industrial University-International Joint Research Center for Persistent Toxic Substances (IJRC-PTS) within 48 h of sampling and stored at −20 °C until extraction. The subsequent experiments for concentration and composition analysis were completed within one month after sampling.

The main work for sample processing, detection, and analysis were conducted at IJRC-PTS. The Soxhlet’s extraction was applied in the process of sample pretreatment. Briefly, the collected samples were spiked with PCB-155 as a recovery standard and were Soxhlet-extracted with a mixture of acetone:hexane (1:1, *v*/*v*) and dichloromethane for 24 h, respectively. The extracts were then concentrated up to ~1–2 mL using a rotary evaporator, and the concentrated extracts were purified and fractionated using active silica gel columns (activeness for 16 h at 130 °C; diameter 10 mm, length 0.4 m), with 70 mL of dichloromethane:hexane (1:1, *v*/*v*). The treated extracts were then concentrated to exactly 1.0 mL using a gentle nitrogen flow (purity 99.999%), and a known amount of BDE-71 was added as an internal standard. The treated samples were stored in chromatographic bottles at −20 °C [19].

### 2.2. Instrumental Analysis

Tri- to hepta-BDEs was determined by Agilent gas chromatography (GC-6890) coupled with a mass spectrometer (MS-5975), using an electron capture negative ionization (ECNI) ion source in the selected ion monitoring (SIM) mode, equipped using HP-5 MS capillary column (30 m × 0.25 mm × 0.25 μm, J&W Scientific, Folsom, CA, USA). The ion source was held at 200 °C, and the GC to MS transfer line was at 280 °C. Initial oven temperature was set at 110 °C and held for 1 min, followed by an initial ramp of 20 °C/min to 250 °C held for 5 min and a second ramp of 25 °C/min to 300 °C for a total run of 15 min. The injection volume of each sample was 2.0 μL under split less mode. Helium was used as a carrier gas at a flow velocity of 0.8 mL/min, and methane was used as the chemical ionization moderating gas.

BDE-209 was determined by Varian CP-3800 gas chromatography coupled with an electron capture detector (GC/ECD), equipped with a 7 m (250 μm i.d., 0.25 μm film thickness; J&W Scientific, Folsom, CA, USA) HP-5 MS capillary column. The column oven temperature program was: 100 °C held for 2 min, then 6 °C to 210 °C held for 5 min, and 7 °C to 300 °C held for 10 min; injector: 250 °C; and detector: 310 °C. Nitrogen was used as the carrier gas at a constant flow of 2.0 mL/min [19].

The concentration of BDEs was measured using quantitative analysis by applying the internal standard (BDE-71) method. Species identification was achieved by comparing the mass spectra and retention times of the chromatographic peaks with the corresponding and authentic standards [19,20].

### 2.3. Quality Assurance/Quality Control

The pretreatment and measurement process was strictly carried out following the IJRC-PTS quality control method system. Blanks were added for every 12 samples prepared by loading a PUF plug and GFF filter to the sampler for 24 h during the sampling period with no air passing through. As a result, the blank levels were less than 20% of the sample’s mass, and they were appropriately subtracted from the sample.

The gaseous phase spike test was taken once a month, using a second PUF plug following the first PUF plug, with second PUF plug divided into two layers (2 cm + 3 cm in thickness). PBDE contents in the last plug were 5% less than those in the first plug for all samples but BDE-85, which yielded an average of 8%.

The instrument detection limits (IDL) for PBDEs ranged from 0.05 to 0.83 ng/mL. If the concentrations of congeners were below IDL, we recorded it as “0”.

### 2.4. Clausius–Clapeyron Equation

Previous studies had shown that the atmospheric partial pressures of legacy semi-volatile organic compounds strongly correlate with atmospheric temperature [15,21,22], and this relationship could be described using the Clausius–Clapeyron (C–C) Equation (1):

(1) $$\ln P=\frac{-∆H}{R}\times \frac{1}{T}+const.$$

(2) $$\mathrm{PV}=\mathrm{nRT}\to P=\frac{m}{M}\times \frac{1}{V}\times RT\to \ln P=\ln\left(\frac{c}{M}\times RT\right)$$

where P is the partial pressure in atm that could be obtained from the concentration according to gas formula of state (2); ∆*H* is the heat of vaporization in kJ/mol; R is the gas constant; *T* is the ambient temperature in K; *n* is the amount of substance; *m* and *V* are mass and volume, respectively; *c* is the concentration; and *M* is the molar mass.

This equation relates the increase in natural logarithm vapor pressure to the increase in temperature and predicts that these compounds could re-volatilize from terrestrial and aquatic surfaces more readily as temperatures increase. Meanwhile, this study introduces the concept of relative natural logarithm of partial pressure (RP) [23], in order to evaluate the degree of deviation between the measured natural logarithm of partial pressure (ln *P*) and the predicted natural logarithm of partial pressure (ln *P*_0_). Specifically, RP = ln *P* – ln *P*_0_. Half the value of the RP standard deviation (1/2*S_RP_*) was defined as the category boundaries (as shown below), which were further analyzed below.

(3) $$\{\begin{array}{c} RP_{i}\leq -\frac{1}{2}S_{RP}\to RP_{i}\epsilon Low\\ -\frac{1}{2}S_{RP}\leq RP_{i}\leq -\frac{1}{2}S_{RP}\to RP_{i}\epsilon Middle\\ RP_{i}\geq \frac{1}{2}S_{RP}\to RP_{i}\epsilon High \end{array}$$

### 2.5. Hybrid Single-Particle Lagrangian Integrated Trajectory (HYSPLIT) Model

The HYSPLIT model is a well-developed system for computing simple air parcel trajectories, as well as complex transport, dispersion, chemical transformation, and deposition simulations. HYSPLIT continues to be one of the most extensively used atmospheric transport and dispersion models in the atmospheric sciences community [24].

Therefore, the HYSPLIT model was applied in our study to investigate the long-distance transport impact on PBDEs in Xi’an city, NW China. The meteorological data were accessed from the Weather Underground [25], and 96 h back trajectories originated at 00:00, 08:00, and 16:00 of each sampling day were calculated using the HYSPLIT, which resulted in 402 tracks. The arrival height of the trajectories was 500 m above ground level, which was shown to reflect the average flow field characteristics of the boundary layer [26].

### 2.6. Cluster Analysis

The clustering method based on airflow trajectory was applied to classify large numbers of trajectories based on spatial similarities (i.e., transmission speed and direction) of airflow [27]. In order to understand the transport path of main pollutants within the research area, the Ward’s variance method was used in our study. At the same time, the angle distance algorithm was used to classify different types of airflow [28], and, based on the results of the above-mentioned analyses, the pollutant concentration characteristics corresponding to each group of airflow were investigated.

### 2.7. The Concentration Weighted Trajectory (CWT)

The CWT method could easily distinguish source strength by assigning the concentration values at the receptor site to their corresponding trajectories [29]. In the CWT method, a grid is superimposed over the trajectory computations’ domain. Each grid cell is assigned a residence time weighted concentration from the measured sample associated with the trajectories that crossed that grid cell as follows:

(4) $$C_{ij}=\frac{1}{\sum_{l=1}^{M}\tau_{ijl}}\sum_{l=1}^{M}C_{l}\tau_{ijl},$$

where *C_ij_* is the average weighted concentration in the *ij*th cell, l is the index of the trajectory, *M* is the total number of trajectories, *C_l_* is the concentration observed on arrival of trajectory *l*, and *τ_ijl_* is the time spent in the *ij*th cell by trajectory *l*.

The time a trajectory spends in a cell could be represented by the number of trajectory segments located in the cell. Furthermore, the concentrations could be transformed to their logarithmic value if less weight is desired for high concentration outliers. A high value for *C_ij_* implies that air parcels traveling over the *ij*th cell would be, on average, associated with high concentrations at the receptor.

A grid cell of 0.5 × 0.5 latitude and longitude was used in this study. Generally, CWT results showed possible source areas instead of indicating individual sources due to the trailing effect, plume dispersion, and inherent trajectory inaccuracies. The trailing effect occurred because CWT gives a constant weight along the trajectory path. Therefore, an arbitrary weighting function (*Wij*) was applied to CWT values to reduce uncertainty in grid cells with a small number of endpoints, since a small number of endpoints in a grid cell (*nij*) resulted in CWT values with high uncertainty. The function used was:

(5) $$W\left(n_{ij}\right)=\{\begin{array}{cc} 1.00 & 2\times ave<n_{ij}\\ 0.75 & ave<n_{ij}\leq 2\times ave\\ 0.50 & 0.5\times ave<n_{ij}\leq ave\\ 0.15 & n_{ij}\leq 0.5\times ave \end{array},$$

where “*ave*” is the average number of endpoints per each cell.

## 3. Results and Discussion

### 3.1. Concentration and Congener Profiles

#### 3.1.1. Residue Levels 

Twelve PBDE congeners (BDE-17, -28, -47, -66, -85, -99, -100, -138, -153, -154, -183, and -209) were detected in the collected samples. Concentrations and detection frequencies of both gaseous and particulate phases are summarized in Table 1 for the above mentioned PBDEs congeners.

The data show that the higher brominated congeners such as BDE-99, -100, -154, -183, and -209 possessed higher detection frequencies (>90%), and their concentrations were higher in particulate phases compared to those in gaseous phase. Nevertheless, concentrations of the lower brominated congeners, like BDE-17, -28, and -47, which were frequently detected in the gaseous phase, were higher in gaseous phase compared to the particulate phase.

The concentration of Σ_12_PBDEs were 4.79 ± 5.59 pg/m^3^ (dry weight, same below) and 248.4 ± 199.0 pg/m^3^ in the gaseous and particulate phases, respectively. Σ_12_PBDEs total concentration of both gaseous and particulate phases was 253.2 ± 198.4 pg/m^3^, ranging from 36.4 to 1055 pg/m^3^ during the whole sampling period.

Interestingly, total concentrations of PBDEs—excluding BDE-209—were very low, ranging from 2.26 to 85.94 pg/m^3^ with an average concentration of 20.64 ± 19.74 pg/m^3^. Although BDE-209 was detected only in the particulate phase, it was the dominate congener in all collected samples, with a much higher concentration (232.6 ± 193.0 pg/m) and a contribution rate of 88.75 ± 13.29% of total atmospheric PBDEs.

The seasonal average concentrations (both gaseous and particle phase) of BDE-209 and Σ_11_PBDEs during the sampling period were 266.4 ± 197.3 and 322.5 ± 200.6 pg/m^3^ in summer; 188.8 ± 173.8 and 88.34 ± 74.90 in spring; 16.13 ± 16.26 and 14.45 ± 9.20 in autumn; and 25.31 ± 22.53 and 24.21 ± 22.70 pg/m^3^ in winter, respectively. The highest concentration of Σ_11_PBDEs (averaged 53.65 pg/m^3^) during winter was measured in 2008, meanwhile the lowest value was obtained in 2010 (averaged 12.28 pg/m^3^). The average concentration of BDE-209 in autumn 2008 was higher than that in other sampling years.

**Figure 1:**
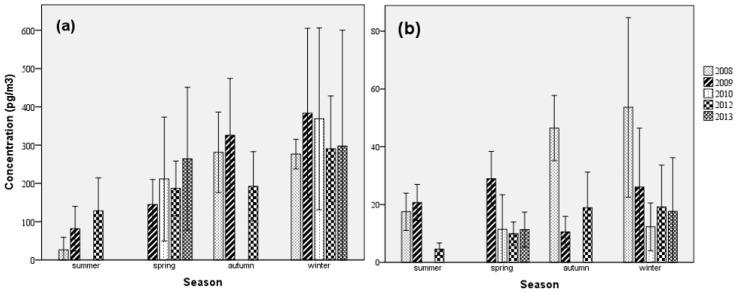
Annual and seasonal average concentrations (pg/m^3^) of BDE-209 (**a**) and Σ_11_PBDEs (**b**).

There were some differences in the annual concentration variations of Σ_11_PBDEs in each season as shown in Figure 1b. The highest Σ_11_PBDEs concentrations (44.04 ± 21.61 pg/m^3^) were measured in 2008, which were over twice those during other sampling years. On the contrary, the annual concentration variations of BDE-209 were not as obvious (Figure 1a). In descending order, BDE-209 concentrations were as follows: 2013 (282.8 ± 187.6 pg/m^3^), 2010 (261.1 ± 260.4 pg/m^3^), 2008 (243.7 ± 163.4 pg/m^3^), 2009 (230.7 ± 212.0 pg/m^3^), and 2012 (206.5 ± 156.0 pg/m^3^). We found that the BDE-209 concentrations in the same season of every sampling year were similar to each other, since deca-BDE was not yet banned and had been perpetually widely used, it was detected frequently in the atmosphere year by year due to its stable chemical property.

Furthermore, it should be noted that the sampling period was discontinuous, and seasonal concentrations of some years (2008, 2010, and 2013) were not included, except in the winter, which we sampled each year and thus may interfere with the results.

#### 3.1.2. Congener Profiles

Although a large amount of research has focused on atmospheric PBDEs in different areas around the world, it has been difficult to perform a systematic comparison of these studies due to the inconsistencies in the number of congeners, the types of samples, and the sampling periods. In order to further analyze and investigate the concentration level of PBDEs in this study, we performed a simple comparison based on concentration distribution (Table 2). The data show that the concentration of BDE-209 could equal ten times the sum of other target PBDE congeners, and that BDE-209 dominated the congeners in most collected samples. Actually, this result was not unexpected since BDE-209 is the only congener of commercial deca-BDE that contributes more than 80% to the total PBDE production [41]. The ambient air around Binhai’s economic development zone in Weifang [39], the largest manufacture base of BFRs in China, had the highest ever-reported atmospheric concentrations of BDE-209 (averaged 1.4 × 105 pg/m^3^). This value was up to six hundred times higher than that in our study.

Interestingly, the ratio for BDE-209 to total PBDE in Guiyu’s ambient air was ~54.3% [33], which was lower than that in other areas. Guiyu is the largest electronic waste dismantling area in China, where penta-BDE replaced deca-BDE in the main electronic products, which inevitably would permeate into water, soil, and atmospheric environment, the main route of transmission [41]. As a result, the concentration of PBDEs in Guiyu’s air was 9579 pg/m^3^, while the concentration of BDE-209 was only 2164 pg/m^3^, but both values were higher than those in other cities.

Overall, PBDE pollution levels in Xi’an were relatively high compared to areas abroad, i.e., Chicago [30], Izmir [32], and Athens [36], but were much milder than those of southeast coastal cities in China, such as Guangzhou [31], Taizhou [35], and Shanghai [34]. These data indicate that Xi’an, located in Midwest China, exhibited a moderate PBDE pollution level compared to other cities like Beijing [37], Nanning [39], and Wuhan [40]. Furthermore, concentration levels of ΣPBDEs^b^ and BDE-209 in Xi’an city were more similar to those of Chaohu Lake [38], located in the central part of China, and the sampling experiments of these two studies were almost conducted at the same time.

### 3.2. Temperature Dependence and Cluster Analysis

**Figure 2:**
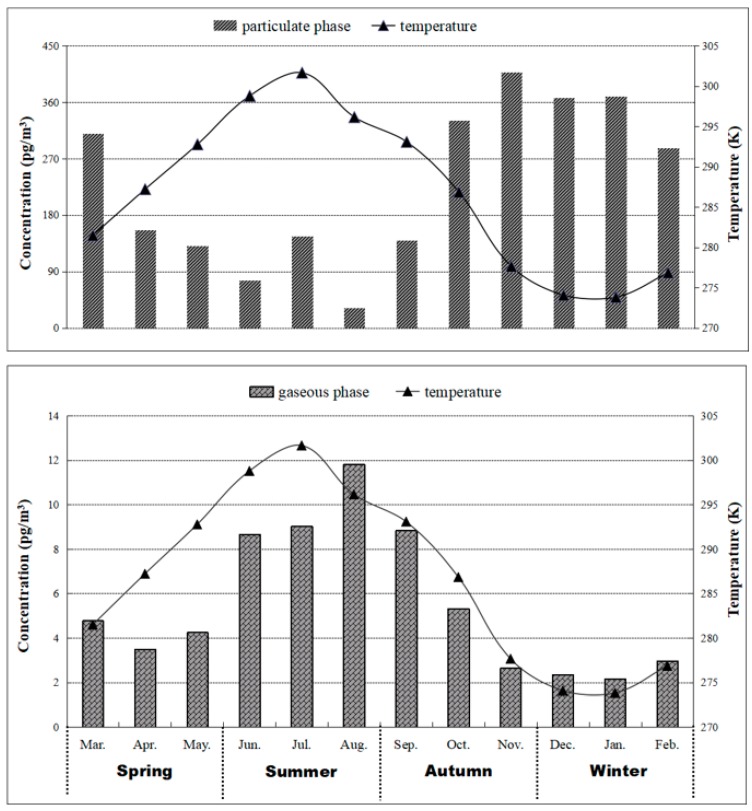
The distribution trend of monthly mean concentration and monthly average temperature.

As shown in Figure 2, Σ_12_PBDEs (particulate phase) concentrations during winter were the highest, followed by those in autumn, and the lowest were during summer. By contrast, the gaseous phase concentrations of Σ_12_PBDEs were significantly higher during the summer compared to the other three seasons, especially winter, which had a minimum value of gaseous Σ_12_PBDEs.

**Figure 3:**
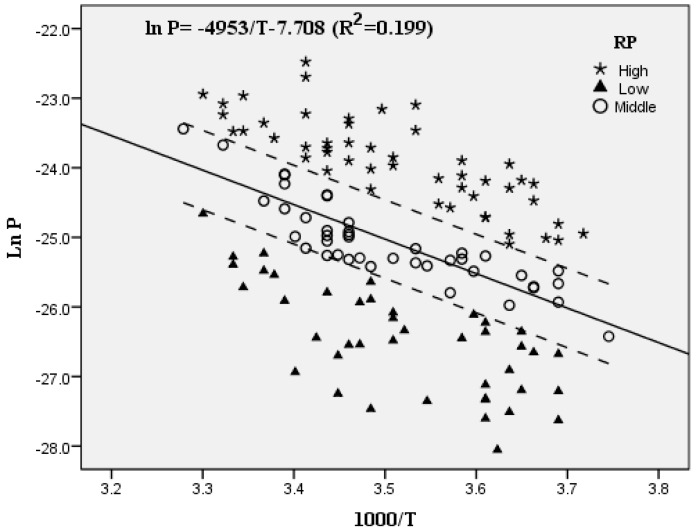
Regression plots and R^2^ of the Clausius–Clapeyron plots (natural logarithms of partial pressure of PBDEs vs. reciprocal temperature) of PBDEs (gaseous phase).

Obviously, PBDE concentrations in particulate and gaseous phases exhibited opposite trends with the variation in temperature. More specifically, particulate PBDE concentrations decreased gradually as the temperature increased. By contrast, gaseous PBDE concentrations varied consistently with the temperature. Furthermore, significant negative correlations (*p* < 0.05) between lnP and 1/T were observed for most compounds that exited in the gaseous phase. BDE-209 was not considered because it was only observed in the particulate phase. The linear regression equation was obtained between partial pressures and ambient temperature, which was ln P = −4953/T−7.708 *(R^2^* = 0.199), as shown in Figure 3.

The collected samples were divided into three groups: low RP, middle RP, and high RP, according to the differences in RP values. The number of each group was 42 (low RP), 42 (middle RP), and 50 (high RP). The linear regression analysis was further conducted on the basis of the three RP groups (high, low, and middle), the R^2^ was 0.662, 0.495, and 0.750, respectively, which indicated that the results of linear regression were acceptable.

In addition, sampling days were divided into categories according to four main factors including temperature, wind speed, precipitation, and wind direction, because different meteorological conditions could influence the volatilization and diffusion of PBDEs, as shown in Table 3.

For example, if the sample receives more than 1.0 mm of precipitation per 24 h, it was regarded as a precipitation sample, otherwise it was regarded as a no-precipitation sample. The demarcation criterion between low and high temperatures was the local annual average temperature (12.08 °C). Wind speed was determined using 96 h back-trajectory maps. If wind blew at less than 800 km over 96 h (about 2.2 m/s: the average speed of all the back trajectories), it was categorized into the slow transport category, and if above this value, the sample was categorized into the fast transport category. Wind direction was classified into two categories, northwest (prevailing wind direction annual) and southeast, based on the source locations upwind from the sample site. To help determine the conditions responsible for a sample belonging to a particular category, back trajectories were calculated for all of the samples using HYSPLIT.

Table 3 shows that samples with low RP always occurred when the temperature was low (54.76% > 45.24%), winds came from the northwest sector (66.67% > 61.19%), and transport speeds were fast (54.76% > 46.27%). Meanwhile, a high PBDEs RP was related to the conditions of no obvious precipitation (82.00% > 79.85%), the wind coming from the southeast (42.00% > 38.81%), and high temperature (54% > 50.75%). In addition, middle RP occurred on days with low wind speeds (61.90% > 53.73%) and rainfall (23.81% > 20.15%).

**Figure 4:**
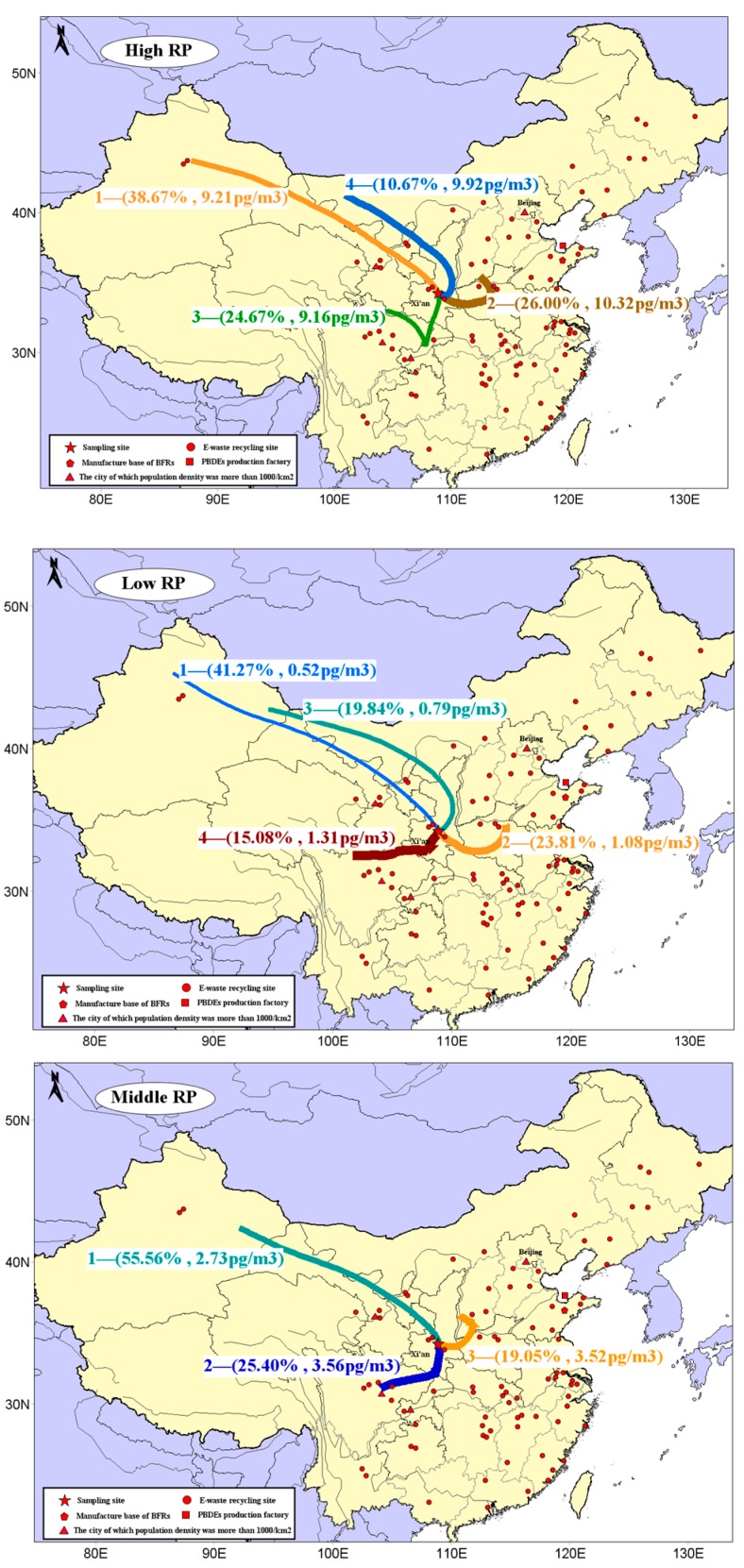
Clustering analysis results under different RP values (note: trajectory thickness was related to the level of PBDE concentration it corresponds to).

Moreover, in order to investigate the relationship among meteorological factors, airflow trajectory, and concentration levels of gaseous PBDEs, the cluster analysis was carried out when the RP values were low, middle, and high (Figure 4).

When the RP value was low, there were four types of trajectories. The two trajectories that came from the northwestern direction accounted for high probability (61.11% in total) but with low concentration levels (0.52 pg/m^3^ and 0.79 pg/m^3^). Meanwhile, concentrations (1.31 pg/m^3^ and 1.08 pg/m^3^) of southeastward trajectories were twice those of the other two trajectories, although with low probability (38.89% in total). This could be due to the fact that the common low temperature was not conducive to pollutant volatilization under low RP, while the fast northwest wind brings a large amount of fresh air from relatively unpolluted areas with significant dilution to the sampling site.

In contrast, among the four trajectory types analyzed at higher RP values, the probability of two types of trajectories occurring from the southeast was higher than that from the northwest, and the corresponding pollution concentration levels were relatively high at 10.32 pg/m^3^ and 9.16 pg/m^3^, respectively. We also found that the trajectories from the southeast were short. We speculate that the high temperature and dry weather conditions were not conducive of pollutant diffusion, because there are many pollution sources in the southeast area in Xi’an city, and the high-frequency of southeast wind could easily bring pollutants into the atmospheric environment of the sampling point.

In addition, three types of trajectories were analyzed at middle PR levels, among which the trajectories from the northwest had the highest probability of occurrence (55.56%), followed by the trajectories from the southwest (25.40%), and then trajectories from east to north (19.05%). The concentration levels corresponding to the three kinds of trajectories were 2.73 pg/m^3^, 3.56 pg/m^3^, and 3.52 pg/m^3^, respectively. These data indicated that the concentrations of trajectories with less proportions were significantly higher than those with higher proportions. In addition, comparing track characteristics at high and low RP values showed that the length of the track path at middle RP value was much shorter, which could indicate that at middle RP value, the PBDEs in the atmospheric environment in Xi’an might be dominated by local sources.

### 3.3. Potential Source Identification

#### 3.3.1. Principal Component Analysis (PCA)

For a better illustration of PBDE compositions and sources in Xi’an’s atmospheric environment, the PCA and maximum variance rotation method were used to analyze the aforementioned PBDE congeners in all collected samples. Four main factors, whose eigenvalues were greater than “1”, were extracted. The cumulative variance contribution rate of these four factors was 84.38%, which could better reflect the total information of the original variables. The concrete results are shown in Table 4.

Table 4 shows that the variance contribution rate of factor 1 was 32.74%. The components with higher load were BDE-17, -28, -47, -99, and -153. Since BDE-47, -99, and -153 were the main components of penta-BDE, while BDE-17 and -28 were the precursor substances of BDE-47 [42], factor 1 could be the product of penta-BDE. Components with a higher load in factor 2 were BDE-66, -100, -154, and -183. Since BDE-183 was representative of octa-BDE [43], factor 2 might be commercial octa-BDE. In factor 3, BDE-85 and -138 exhibited higher loads, and the variance contribution rate was 17.7%. This factor could be regarded as commercial penta-BDE [43]. The percentage variance of factor 4 was 12.9%, and the highest load was BDE-209, which was the main industrial product of deca-BDE. In summary, the most likely sources of PBDEs in Xi’an’s atmospheric environment are commercial penta-BDE, followed by octa-BDE, and deca-BDE industrial products. In this study, PCA analysis results were different from those in other regions of China, such as Wuhan [40], Heilongjiang [44], and Chaohu Lake [38].

#### 3.3.2. CWT Modeling

Potential PBDE source regions at Xi’an sampling sites were further identified by calculating the four-day (96 h) back-trajectories of air masses at altitudes above 500 m once every 12 h in 2008–2013 using the HYSPLIT model.

Related studies have shown that PBDEs of deca-BDE, and other particulate phases, attenuated in the form of dry and wet deposition during transport, while gaseous phase PBDEs were rarely affected by dry and wet depositions. Therefore, this paper mainly focuses on gaseous phase PBDEs to analyze the potential pollution source region [9].

**Figure 5:**
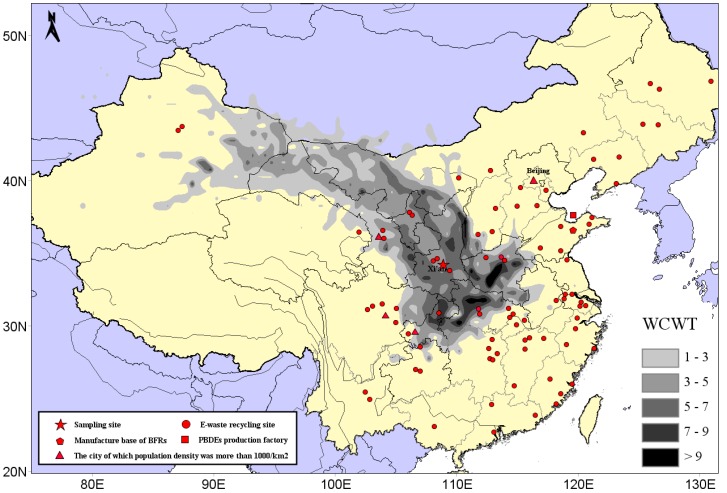
CWT results of gaseous PBDEs with four-day back-trajectories from 2008 to 2013. The pentagram is the sampling site, red dots are E-waste recycling site, and the triangles are the cities whose population density was more than 1000/km^2^. Notes: the dots in the figure represent the E-waste recycling sites, which were available online from the Ministry of Environmental Protection website [45].

In order to further determine the possible PBDE sources in Xi’an’s atmosphere, CWT analysis of PBDEs (gaseous phase) was carried out. In Figure 5, the distribution diagram of CWT showed the contribution of potential source areas to the concentration of PBDEs in Xi’an’s atmosphere. Darker colors indicate greater contribution of the source area.

As shown in Figure 5, Henan province, the southwest and northwest areas of Hubei province, have the darkest colors, and their corresponding average daily contributions to PBDEs were the highest respectively, above 9 pg/m^3^. Zhengzhou (in Henan) and Jingmen (in Hubei) were areas for electronic waste dismantling, so the PBDEs existing in these locations were a potential source of PBDE pollution in Xi’an’s atmospheric environment. Furthermore, the border colors between Shaanxi and Shanxi, Shaanxi and the northern part of Chongqing, and Shaanxi and the southern part of Henan, were relatively dark, and their daily contribution to local PBDE pollution was ~7–9 pg/m^3^. Parts of Gansu, Inner Mongolia, Sichuan, Xinjiang, and Jiangsu also contributed 3–7 pg/m^3^ to the receiving point on a daily basis. It should be noted that the regions mainly including northeast Xinjiang, northwest Inner Mongolia, and the Gansu and Ningxia regions remain relatively underdeveloped in terms of economy and social development, they are sparsely populated, and have no obvious E-waste recycling sites. These areas were located just in the upwind direction of our sampling points during our research and were therefore likely to be identified as the source regions due to the “trailing effect”. CWT results (daily contribution of 1–3 pg/m^3^) of these regions may not be accurate enough.

## 4. Conclusions

Twelve kinds of PBDE congeners were detected in Xi’an atmospheric samples, and BDE-209 was indicated as the dominate congener due to its extensive use. Principal component analysis revealed that commercial penta-BDE, octa-BDE, and deca-BDE might be the main sources of PBDEs in Xi’an city’s atmosphere. Meanwhile, changes in annual PBDE concentrations did not show a downward trend in this study, although the production and use of PBDEs had been prohibited worldwide since May 2009. Meanwhile, the potential pollution source areas of gaseous phase PBDEs were analyzed using the CWT method. We found that the color of gaseous phase PBDEs in central and southwest Henan Province and northwestern Hubei Province were the darkest. Therefore, these areas were seen as the main potential pollution source.

## Figures and Tables

**Table 1:** The concentration and detection frequency of each polybrominated diphenyl ether (PBDE) congener.

Classify	Congener	Detection Frequencies		Concentration (pg/m^3^)			Proportion
		Gaseous Phase	Particulate Phase	Gaseous Phase	Particulate Phase	Total	
tri-BDE	BDE-17	98.51%	88.06%	1.14 ± 1.15	0.50 ± 1.08	1.64 ± 1.68	9.70% ± 6.10%
	BDE-28	83.58%	70.90%	1.05 ± 1.54	0.48 ± 1.26	1.53 ± 2.16	5.90% ± 5.70%
tetra-BDE	BDE-47	40.30%	47.76%	1.20 ± 1.94	1.43 ± 2.57	2.63 ± 3.50	9.50% ± 10.40%
	BDE-66	59.70%	73.88%	0.20 ± 0.31	0.60 ± 0.66	0.80 ± 0.68	5.70% ± 4.50%
penta-BDE	BDE-85	70.90%	65.67%	0.19 ± 0.21	0.46 ± 1.36	0.65 ± 1.40	5.20% ± 6.10%
	BDE-99	76.87%	97.76%	0.44 ± 0.86	3.51 ± 4.80	3.95 ± 5.03	17.20% ± 7.70%
	BDE-100	76.12%	96.27%	0.17 ± 0.31	1.61 ± 2.42	1.79 ± 2.55	11.80% ± 12.90%
hexa-BDE	BDE-138	35.82%	51.49%	0.12 ± 0.62	1.28 ± 3.19	1.40 ± 3.32	6.30% ± 9.90%
	BDE-153	54.48%	82.84%	0.12 ± 0.25	3.16 ± 3.92	3.28 ± 3.90	15.00% ± 9.20%
	BDE-154	58.96%	95.52%	0.11 ± 0.20	1.22 ± 2.34	1.32 ± 2.33	7.30% ± 4.80%
hepta-BDE	BDE-183	36.57%	90.30%	0.05 ± 0.12	1.23 ± 1.80	1.28 ± 1.81	6.40% ± 3.90%
deca-BDE	BDE-209	0.00%	100.00%	0.00 ± 0.00	232.6 ± 193.0	232.6 ± 193.0	-
	^1^ Σ_12_PBDEs	-	-	4.79 ± 5.59	248.4 ± 199.0	253.2 ± 198.4	-
	^2^ Σ_11_PBDEs	-	-	4.79 ± 5.59	15.8 ± 17.7	20.6 ± 19.7	-

Note: ^1^ Σ_12_PBDEs refers to the sum of all target PBDEs. ^2^ Σ_11_PBDEs refers to total PBDEs excluding BDE-209.

**Table 2:** Comparison of atmospheric PBDE concentrations (pg/m^3^) obtained in this study with those in other regions.

Sampling Site	Year	Arithmetic Mean Concentration		Concentration Range		N ^a^	References
		ΣPBDEs ^b^	BDE-209	ΣPBDEs ^b^	BDE-209		
Chicago, USA	2002–2003	31.6	68.4	9.6–68	2.6–956	19	[30]
Guangzhou, China	2004	1024	1423	88.8–3673	263.8–4200	11	[31]
Izmir, Turkey	2004–2005	31.4	30.7	-	-	7	[32]
Guiyu, China	2005	9579	2164	-	-	11	[33]
Shanghai, China	2006	104 ± 54	640 ± 143	-	-	20	[34]
Taizhou, China	2006–2007	79.4	210.5	17–165	86–439	13	[35]
Athens, Greece	2006–2007	13	-	21–30	-	12	[36]
Beijing, China	2009–2010	6.2	164	nd ^c^–23.6	30.7–454	8	[37]
Lake Chaohu, China	2010–2013	15.4	233.9	2.2–72.0	nd–233.9	14	[38]
Weifang, China	2011–2012	228	1.4 × 10^5^	-	1.5 × 10^4^~2.4 × 10^5^	8	[39]
Nanning, China	2011–2012	8.4	314.4	-	27.1~783.0	8	[39]
Wuhan, China	2015–2016	14.5	-	5.99–45.4	-	9	[40]
Xi’an, China	2008–2010	24.8	241.2	2.26–85.94	0–1041	12	This study
	2012–2013	14.28	219.4	2.39–85.15	50.6–764.5	12	This study

Notes: ^a^ Numbers of PBDE congeners—It should be noted that different numbers of target compounds (PBDE compounds) were employed in different studies. ^b^ BDE-209 was not included in the concentrations of ΣPBDEs. ^c^ nd means not detected in experiment.

**Table 3:** Percentage of events that occurred at each criterion of temperature, precipitation, wind direction, and wind speed for PBDEs. The number in the parenthesis reflects the number of samples in each category.

Meteorological Factor			Relative PBDEs Partial Pressure		
			Low (42)	Middle (42)	High (50)
Temperature	High (68)	50.75%	45.24%	52.38%	**54.00%**↑
	Low (66)	49.25%	**54.76%**↑	47.62%	46.00%
Precipitation	Yes (27)	20.15%	19.05%	**23.81%**↑	18.00%
	No (107)	79.85%	80.95%	76.19%	**82.00%**↑
Wind Speed	Fast (62)	46.27%	**54.76%**↑	38.10%	46.00%
	Slow (72)	53.73%	45.24%	**61.90%**↑	54.00%
Wind Direction	Northwest (82)	61.19%	**66.67%**	61.90%	58.00%
	Southeast (52)	38.81%	33.33%	38.10%	**42.00%**↑

Notes: The arrowhead indicates that the probability of occurrence of a weather phenomenon increases under the RP value of this group.

**Table 4:** Total variance interpretation rate of principal component analysis (PCA).

Principal Component	Eigenvalue	Percentage Variance (%)	Cumulative Percentage (%)	PBDEs Congeners with Higher Load
1	3.929	32.74	32.74	BDE-17, -28, -47, -99, -153
2	2.527	21.06	53.80	BDE-66, -100, -154, -183
3	2.124	17.70	71.50	BDE-85, -138
4	1.546	12.88	84.38	BDE-209

Note: Factors with eigenvalues less than “1” were not listed in the table.
